# Crustose Calicioid Lichens and Fungi in Mountain Cloud Forests of Tanzania

**DOI:** 10.3390/microorganisms7110491

**Published:** 2019-10-26

**Authors:** Stella Gilbert Temu, Sanja Tibell, Donatha Damian Tibuhwa, Leif Tibell

**Affiliations:** 1 Department of Organismal Biology, Uppsala University, Norbyvägen 18D, 75236 Uppsala, Sweden; sanja.tibell@ebc.uu.se (S.T.); leif.tibell@gmail.com (L.T.); 2 Department of Molecular Biology and Biotechnology, University of Dar es Salaam, Dar es Salaam 35065, Tanzania; dtibuhwa@yahoo.co.uk

**Keywords:** calicioid, conservation, lichen diversity, taxonomy

## Abstract

A total of 26 crustose calicioid lichens and fungi were found in Tanzania. Most of them belong to a group of species with wide distributions in cool areas of both hemispheres and occasional occurrence in high mountains at low latitudes. In Tanzania calicioids mainly occur in the middle and upper forest zones and their niches are found on the bark of old trees and on lignum, most of them restricted to mountain cloud forests. Calicioids are rare and often red-listed, and are also bioindicators of long forest continuity. Consequently, they form an important biota in mountain cloud forests and deserve attention in the context of preserving biodiversity and developing conservation policies. One new species, *Chaenothecopsis kilimanjaroensis*, is described. *Chaenotheca hispidula* and *Pyrgillus cambodiensis* are reported as new to Africa and *Calicium lenticulare* and *Chaenothecopsis debilis* are reported as new to Tanzania.

## 1. Introduction

Calicioid lichens and fungi represent an artificial, antiquated concept and not a natural grouping of species. Very early on the “Calicioidea” [1,2,3] were recognized as a group characterized by having a mazaedium (mature spores being collected on the surface of the fruit body) and typically they were crustose lichens with stalked fruit bodies. With this circumscription, they were also later to be considered a prime example of a natural group, often ranked as an order, Caliciales [4]. This approach was later codified, e.g., by Keissler [5] as “Coniocarpinae”. The emphasis on the cardinal character of a mazaedium also led to the inclusion of fruticose lichens (e.g., *Sphaerophorus* Pers.). In addition, minute fungi with apothecia superficially similar to those originally included in the “Calicioidea” came to be added, for example, genera like *Chaenothecopsis* Vain. and *Stenocybe* Nyl. ex Körb., both of which later were transferred to Mycocaliciales [6]. That the calicioids are not monophyletic was first suggested by [7] based on morphological and secondary chemistry data, a conclusion later supported by molecular data in later studies [8,9]. The calicioids are thus derived from various ascomycete clades, such as Arthoniales, Lecanorales, and Pyrenulales, and primarily characterized by the parallel development of passive spore dispersal and/or a superficial [7,9] morphological similarity with archetypic “Calicioidea” in which the ascomata are differentiated into a stalk and distinct capitulum [8]. The ascomata accumulate mature spores on the surface of the fruit bodies as a thick, often black or brown layer, the mazaedium, and unlike most ascomycetes the spore dispersal is passive [7,8]. Calicioids, mazedium-forming and non-mazaediate alike, are often found together in similar habitats, mostly on bark and wood in undisturbed primary forests [10,11]. Calicioids are frequently used in nature conservation as bioindicators of species-rich areas with long forest continuity [11,12]. They are mainly rare and threatened by habitat destruction—usually forest management—and, hence, often included in Red lists [13].

Forest biodiversity conservation in Tanzania covers 38% of the total land area on the mainland and is made up of about 33.5 million hectares of forests and woodlands [14]. Cloud montane forest area is limited and cloud forests are characterized by the frequent incidence of fog, low clouds [15], and dew formation [16]. They play an important role in filtering water pollutants, regulating water yield and flow, moderating floods, and enhancing precipitation by capturing moisture from clouds [14,17]. Cloud montane forests harbor a wealth of rare and threatened species and, in addition, the forests play a vital role in the provision of freshwater [15,18]. The Kilimanjaro National park forest has a high biodiversity and a low degree of endemism. The forest comprises several altitudinal zones such as the colline (lowland) zone; the submontane zone; the lower (elevation 1800–2200 m), the middle (2200–2500 m), and upper (2500–3850 m) montane zones, and the subalpine forest zone (4000 m and above) [19,20]. Forests of the middle and upper montane zones and subalpine zone can be defined as “cloud forests” since they are usually covered by fog [20]. Forests of the lower montane zones harbor a mixture of *Ocotea usambarensis*, *Agarista salicifolia*, *Macaranga capensis* var. *kilimandscharica*, *Syzygium guineense*, and *Polyscias fulva*. *Ocotea usambarensis* dominates in the middle montane zone. *Podocarpus latifolius*, *Hagenia abyssinica*, and *Prunus africana* dominate the upper montane zone and *Erica trimera* characterizes the subalpine zone [20]. The Monduli forest reserve (Arusha) is well known for harboring a high biodiversity. The forests visited in this study, i.e., Kilimanjaro and Arusha have shown to provide suitable habitats for calicioid lichens in Tanzania.

Calicioid lichens have been widely studied, for example, in Africa and Madagascar [21], Europe [22], North America [23,24], Australasia [25], Central [26], and South America [27,28], and Asia [29,30,31,32,33]. Morphology, secondary chemistry, anamorph data, and molecular data have been used to characterize them [6,7,34]. Investigations of calicioid lichens and fungi in Africa have been rather few, and were mainly published over the past few decades. Some species, such as *Schistophoron tenue* Stirton [35], *Calicium leucinum* Müll. Arg. [36], *Chaenotheca brunneola* var. *africana* Zahlbr. [37], and *Tylophoron africanum* Vain. = *T. moderatum* [38], are represented in early reports from Africa, whereas a more recent contribution [39] describes species new to Africa, based on specimens collected by Lambinon, Swincow, and Santesson in Burundi, Rwanda, and Zaire, Kenya, Tanzania, Uganda, Ivory Coast, and Kenya. A review of the calicioids in Africa [21] showed most species to occur in high mountains of Northern and Central Africa and in the humid parts of South Africa. In subsequent papers [40,41] additional new records from Tanzania were included.

This paper aims to summarize earlier information on the occurrence of crustose calicioids in Tanzanian mountain cloud forests and includes new discoveries from field work in 2018 with observations on the habitat ecology and distribution of the species.

## 2. Materials and Methods

### 2.1. Sampling

Specimens studied were collected jointly by Stella Temu, Sanja Tibell, and Leif Tibell in the Kilimanjaro and Arusha regions, Tanzania, in 2018, with the abbreviation SGT referring to collections by the first author kept in University of Dar es Salaam (UDSM). Additional records were derived from previously collected material, mostly by Andreas Frisch Uppsala University (UPS) and by Vagn Alstrup (UDSM).

### 2.2. DNA Extraction, PCR Amplification, and Sequencing

Total DNA was extracted from freshly collected material temporarily kept at −20 °C using the DNeasy Plant Mini Kit (Quiagen, Hilden, Germany), following the manufacturer’s instructions. About 10 to 30 apothecia, visually free from contamination, were carefully selected under a dissecting microscope and used for DNA extraction. Diluted DNA (10×) was used for PCR amplifications of the Internal transcribed spacer (nuITS) region. Primers ITS1f [42] and ITS4 [43] were used. The AccuPower PCR PreMix (Bioneer, Daejeon, Korea) was used, adding 3 µL diluted DNA, 1.5 µL of each primer (10 mM), and water to a total volume of 20 µL. The PCR protocol was: initial denaturation for 4 min at 95 °C, followed by 35 cycles of 1 min at 94 °C, 1 min at 54 °C, 45 s at 72 °C, and final elongation for 5 min at 72 °C. The PCR products were visualized by electrophoresis on 1.5% agarose gels. Products were purified using Illustra™ ExoStar buffer diluted 10×, following the manufacturer’s protocol. Sequencing was conducted by Macrogen (Available online: www.macrogen.com).

### 2.3. Alignments and Phylogenetic Reconstructions

Five newly generated sequences were aligned along with 37 sequences downloaded from GenBank (Table 1) by using MAFFT v.7 (Available online: https//mafft.cbrc.jp/alignment/server/), after assessment of their quality. The selected DNA sequences downloaded from GenBank, along with the newly produced sequences (Table 1) were assembled and edited using AliView (Available online: https://ormbunkar.se/aliview/) [44].

Phylogenetic relationships and their posterior probabilities (PP) were inferred using a Bayesian approach, and additional support values were estimated using maximum likelihood bootstrap support (MLbs). For the Bayesian analyses, the most likely models of evolution were estimated using the Akaike Information Criterion (AIC) as implemented in Modeltest 3.7 [45]. The GTR + I + G model of evolution was employed.

The Bayesian analysis was executed using MrBayes 3.2.6 [46], where two analyses of two parallel runs were carried out for 10 M generations. Each run included four chains, and trees were sampled every 1000 generations and 25% were discarded as burn in. All runs converged on the same average likelihood score and topology. Maximum likelihood estimates were carried out by RAxML version 8.2.10 using the GTR + G + I model of site substitution [47]. The branch support was acquired by maximum likelihood bootstrapping (MLbs) of 1000 replicates [48], and MLbs ≥ 70% were considered to be significant.

The trees were visualized in FigTree version 1.3.1 [49].

### 2.4. Morphological and Chemical Studies

The morphological study of the thallus and the fruit body was performed on freezing microtome sections 10 to 15 μm thick and on squash preparations under the microscope. The sections were mounted in cotton blue and glycerin.

The color reactions of the thallus and apothecia was tested using a 10% aqueous potassium hydroxide solution (K).

## 3. Results

### List of Crustose Calicioid Species in Tanzania

***Calicium chlorosporum* F. Wilson**

First reported from Tanzania by [21] and later also by [41], but so far only known from high altitudes in the Arusha region.

**Habitat**: At the base of old *Podocarpus* in montane cloud forest.

**Distribution**: A subtropical species described from Australia but widely distributed also in mountainous areas of Africa with occurrences in Ethiopia, Kenya, Madagascar, the Mascarene Islands, South Africa, and Uganda. Additionally reported from Asia (India and Nepal), Australasia [25], and North America.

***Calicium diploellum* Nyl.**

Reported from Tanzania, Morogoro Region, (Uluguru Mts, Luhangalo) by [40].

**Habitat**: On bark in montane cloud forests.

**Distribution**: Earlier reported from Kenya by [21]. Additionally known from Europe.

***Calicium hyperelloides* Nyl.**

**Specimens examined**: **Tanzania**, Kilimanjaro National Park, Marangu route near Mandara hut, 3°05’22.45” S 37°14’37.07” E, 2870 m, SGT 430; 3°05’51.83” S 37°14’14.66” E; 2601 m, SGT 435 (UPS).

**Habitat**: On wood and bark in montane cloud forests.

**Distribution**: First reported from Tanzania by [39] as occurring in the Nguru Mts. in the Morogoro region. Subsequently [21] also reported from the Uluguru and Usambara Mts. Further records from Tanzania: Iringa Region (Idete, Udzungwa Mts, Massisiwe); Morogoro Region (Uluguru Mts, Luhangalo) were given by [40] and in addition from the Iringa region, Udzungwa Mts., and Kilimanjaro region (Mt. Kilimanjaro) by [41]. The species is widely distributed in tropical to warm temperate areas in Europe, Asia, the Americas and Australia and/or Asia.

***Calicium lenticulare* Ach.**

**Specimens examined**: **Tanzania**, Kilimanjaro National park, Marangu route near the Mandara hut, 3°05’51.83” S 37°14’14.66” E, 2601 m, SGT 437; SGT 433 (UPS).

**Habitat**: On wood and bark in montane cloud forests.

**Distribution**: Widely distributed in temperate to warm temperate areas. Known from the Americas, Asia, Australasia and Europe. Previously reported from Burundi [39] as *C. subquercinum* Asah. and from Kenya [50]. New to Tanzania.

***Calicium pluriseptatum* Tibell**

**Specimens examined**: **Tanzania**, Iringa Region, Udzungwa Mts., between Kiwalamo and Idunda, 08°07’50’’ S, 36°04’20’’ E, 1600 m, 1999 Frisch Tz3533 (hb. Frisch).

**Habitat**: *Calicium pluriseptatum* is found at moderate elevations in undisturbed mountain rainforests, where it grows among mosses on bark.

**Distribution**: Only known from Tanzania and Madagascar.

**Note**: *Calicium pluriseptatum* was first described from the Udzungwa Mts. [41], and was also in the same paper reported from Madagascar.

***Calicium salicinum* Pers.**

**Habitat**: On wood and bark in montane cloud forests.

**Distribution**: First recorded from Kilosa and Morogoro Districts (Morogoro Region) of Tanzania [39], and subsequently from Arusha [21] and Massisiwe (Morogoro Region) by [40].

***Chaenotheca chloroxantha* Tibell**

**Specimens examined**: **Tanzania**, Arusha, Monduli forest reserve, 3°14’47.71” S 36°29’22.96” E, 2456 m; SGT 327a; SGT 330; SGT 332; SGT 338a; SGT 340; SGT 379a (UPS). Kilimanjaro National Park, Marangu route near Mandara Hut, 3°05’51.83” S 37°14’14.66” E, 2601 m, SGT 434 (UPS).

**Habitat**: Occurring on trunks of podocarps and *Xymalops monospora* in the upper cloud forest region.

**Distribution**: Hitherto only known from Africa and previously reported from Kenya, South Africa, and Tanzania (Arusha) [21].

***Chaenotheca confusa* Tibell**

**Habitat**: On the barks in Mt Meru forest.

**Distribution**: In Tanzania it was firstly recorded in the South West slope of Mt. Meru [21]. Known from South America.

***Chaenotheca deludens* Tibell**

**Specimens examined**: **Tanzania**, Kilimanjaro National Park, Marangu route near Mandara hut, 3°05’51.83” S 37°14’14.66” E, 2601 m, SGT 432 (UPS).

**Habitat**: Collected on trunks of podocarps in the upper cloud forest region.

**Distribution**: It was first reported from Africa (Tanzania, Kilimanjaro Region, Mt. Kilimanjaro) [39]. Widely distributed and also known from Europe, Asia and South America.

**Note**: *Chaenotheca deludens* belongs to group of species closely related to *C. stemonea*. Originally described from New Zealand, *Chaenotheca deludens* is characterized by having very long-stalked apothecia with flexuous stalks with a slight reddish brown pruina that dissolves in K, rather than forming violet crystals as in *C. gracillima* (Vain.) Tibell and differing from the latter also in lacking an excipulum. The material from Tanzania SGT 432, however, is morphologically quite different. The Tanzanian material has rather short-stalked non-pruinose apothecia with straight stalks and no K+ reaction was observed. Like *C. deludens* the material does have a finely granular thallus and catenulate asci. The Tanzanian material is too scanty for an extensive assessment of its morphology. Although the nuITS of the Tanzanian material in a network analyses of the nuITS is close to *C. deludens* (e.g., to GenBank no. AF408678 from New Zealand, not shown) there are numerous SNPs between them. Material currently referred to *C. deludens* probably form a species complex of morphologically similar species and this should be further studied.

***Chaenotheca furfuracea* (L.) Tibell**

**Specimens examined**: **Tanzania**, Arusha, Meru Forest Reserve, 3°16’58.35” S 36°42’09.41” E, 2096, SGT 420, SGT421, SGT422 (UPS); Monduli forest reserve, 3°14’47.71” S 36°29’22.96” E, 2456 m, SGT 332a (UPS); Kilimanjaro National Park, Marangu route, 3°04’52.24” S 37°10’32.37” E, 2718 m, SGT 428, SGT 431 (UPS).

**Habitat**: Collected on trunks of podocarps and *Nuxia congesta* in the upper cloud forest region.

**Distribution**: First reported from Tanzania (Arusha region) by [21]. Widely distributed in the Northern and Southern Hemisphere.

**Figure 1:**
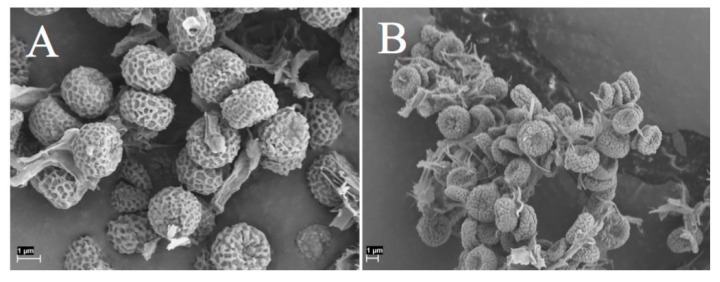
*Chaenotheca furfuracea* spores (**A**) collection from Tanzania, SGT 431. (**B**) collection from Sweden, SGT 441.

**Note**: *Chaenotheca furfuracea* displays intraspecific genetic variation in nuITS sequences. It is characterized by the minutely verrucose spore surface and in this respect Tanzanian material agrees well with material from Europe. (Figure 1). *Chaenotheca confusa* (described from South America) is similar, but has spores with an ornamentation of minute areolae. Considerable variation is, however, found in the nuITS sequences of *C. furfuracea* from various areas (not shown here) and this name may hide several cryptic species.

***Chaenotheca hispidula* (Ach.) Zahlbr.**

**Specimens examined**: **Tanzania**, Arusha, Monduli forest reserve, 3°14’47.71” S 36°29’22.96” E, 2456 m, SGT 389 (UPS); Kilimanjaro National Park, Marangu route near Mandara hut, 3°01’58.59” S 37°10’05.32” E, 2743 m, SGT 425.

**Habitat**: On trunks of podocarps in the upper cloud forest region.

**Distribution**: Widely distributed in Eurasia, the Americas and Australasia. New to Africa.

**Note**: ITS sequences from SGT 389 and SGT 425 differ somewhat between each other and also from sequences available in GenBank under *C. hispidula*. *Chaenotheca hispidula* may thus represent another instance of a complex of morphologically cryptic species in *Chaenotheca* (see also under *C. deludens* and *C. furfuracea*).

***Chaenotheca hygrophila* Tibell**

**Habitat**: On dead wood

**Distribution**: First reported from Tanzania from the Iringa Region (Udzungwa Mts.) [40] and earlier by [51] from “East Africa”.

***Chaenotheca olivaceorufa* (Vain.) Zahlbr.**

**Habitat**: Occurring on trunks of podocarps in the upper cloud forest region.

**Distribution**: First reported from Africa (Tanzania, Kilimanjaro Region, Kilimanjaro) by [40]. Widely distributed in Central and South America and Australasia.

**Note**: This species is closely related to *C. hispidula* and forms part of the critical complex mentioned above under the latter species, and the naming here is provisional. One collection (SGT 425) comes close to material from India and China that may be identified as *C. nepalensis* A. Schmidt, which is similar to *C. olivaceorufa* (described from Brazil) [32] This, however, cannot so far be supported by molecular data since no relevant sequences of South American material is available.

***Chaenotheca sphaerocephala* Nádv.**

**Habitat**: On trunk of old podocarp in rain-shaded situations in mountain cloud forest at 2500–2800 m altitude.

**Distribution**: This species was reported from Mt. Kilimanjaro by [41].

***Chaenotheca stemonea* (Ach.) Müll. Arg.**

**Specimens examined**: **Tanzania**, Kilimanjaro National Park, Marangu route near Mandara hut, 3°01’58.59” S 37°10’05.32” E, 2743 m SGT 424 (UPS).

**Habitat**: On trunk in rain-shaded situations of mountain cloud forest at 2300–2800 m altitude, in Rwanda on *Hagenia abyssinica*.

**Distribution**: From Africa this species was first reported from Rwanda [52], and subsequently also from Tanzania [41].

**Note:** This species is part of the *C. stemonea* complex, and the naming here is provisional since *C. stemonea* in this wide sense may harbor several cryptic species.

***Chaenotheca trichialis* (Ach.) Th. Fr.**

**Specimens examined**: Tanzania, Kilimanjaro National Park, Marangu route near Mandara hut, 3°05’51.83” S 37°14’14.66” E, 2601 m, SGT 438b (UPS).

**Habitat**: On decorticated parts of trunk of *Podocarpus* in mountain cloud forests.

**Distribution**: Reported from the Kilimanjaro Region (Mt. Kilimanjaro) [41] but prior to that reported from Africa also from the DR of Congo, Kenya, and Rwanda [21]. Widely distributed in temperate areas of both the Northern and Southern Hemisphere (Eurasia, Americas, and Australasia). In areas of low latitudes only on high mountains.

***Chaenothecopsis debilis* (Sm.) Tibell**

**Specimens examined**: **Tanzania**, Kilimanjaro Region, Kilimanjaro, Moshi, Mweka Route, 03°10’00’’ S, 37°21’40’’ E, 2700–2900 m, at base of old *Podocarpus* in podocarp mountain forest with *Erica arborea*, 3°10’00’’ S, 37°21’40’’ E, 2700–2900 m, 1999, A. Frisch 99/Tz2783 (hb. Frisch); 03°10’00’’ S, 37°21’40’’ E, 2700–2900 m, 2784 (hb. Frisch).

**Habitat**: In podocarp mountain rainforest at 2700–2900 m.

**Distribution**: Widely distributed in both hemispheres. From Africa previously reported from Algeria [21]. New to Tanzania.

**Figure 2:**
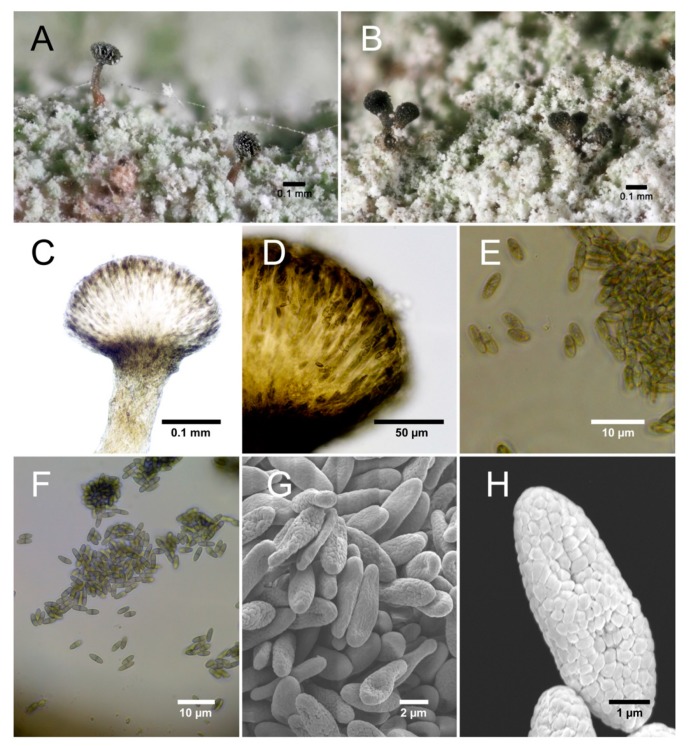
*Chaenothecopsis kilimanjaroensis*; (**A**) Well-stalked apothecia with brown stalks (**B**) aggregated apothecia (**C**); section of apothecium with pale stalk; (**D**) dark brown, well-developed excipulum; (**E**) spores with a faint ornamentation as barely visible under the light microscope; (**F**) spores; (**G**) spores, SEM; (**H**) verrucose spore ornamentation, Scanning Electron Microscopy (SEM).

***Chaenothecopsis kilimanjaroensis* Temu and Tibell sp. nov.** (Figure 2)

*Chaenothecopsis* with a very short stalk which are K-, with single or aggregated capitula formed on the same thallus. The stalk is medium brown towards the base and translucent in water. Spores are uniseptate and uniseriately arranged in asci. Spore ornamentation consisting of elongated blister-like verrucae. Commensal/parasite on sterile lichen crusts and/or the thallus of *Chaenotheca chloroxantha* on bark in montane cloud forests. The attacked host thallus loses pigmentation and turns slightly mauve grey (Figure 2A,B).

Apothecia 0.21–0.27 mm high, very short-stalked or with medium-long, olivaceous brown stalks (Figure 2C). Capitulum single (Figure 2A) and lenticular to broadly obconical, 0.021–0.027 mm diam. (Figure 2A), or 2–5 aggregated capitula (Figure 2B) are formed on the same and then usually short stalk (Figure 2B). Excipulum dark brown (Figure 2D), 6–10 µm thick, consisting of 2–3 layers of periclinally orientated intertwined hyphae measuring 2–3 µm diam. Hypothecium poorly developed, convex, pale with brown/greenish brown hyphae invading from the base. Stalk medium brown, particularly towards the base pale and translucent in water, 0.01 mm diam., consisting of intertwined, periclinal hyphae, K-, Asci cylindrical with uniseriately arranged spores (Figure 2E) and a thickened apex penetrated by a fine canal, 37.4–44.3 µm long and 2.1–2.8 µm wide. Spores (Figure 2F) ellipsoidal to narrowly ellipsoidal, pale brown, 1-septate, 6–6.9 µm long and 2.1–2.6 µm wide, with a rather poorly pigmented septum and a minutely verrucose ornamentation (Figure 2H) barely visible under the light microscope, but under SEM seen to be formed from minute, often slightly elongated blister-like verrucae (Figure 2H).

**Holotype**: **Tanzania**, Arusha, Monduli forest reserve, 3°14’47.71” S 36°29’22.96” E, 2456 m on trunks of *Nuxia congesta*, 16 August 2018, Temu 337 (UPS—holotype; DNA-extraction SGT337, MycoBank no.: MB 833067).

**Additional specimens examined**: **Tanzania**, Arusha, Monduli forest reserve, 3°14’47.71” S 36°29’22.96” E, 2456 m; SGT 327c; SGT 328; SGT 333; SGT 338.

**Habitat**: On trunks of *Nuxia congesta* in montane cloud forest.

**Distribution**: Only known from the type locality.

**Figure 3:**
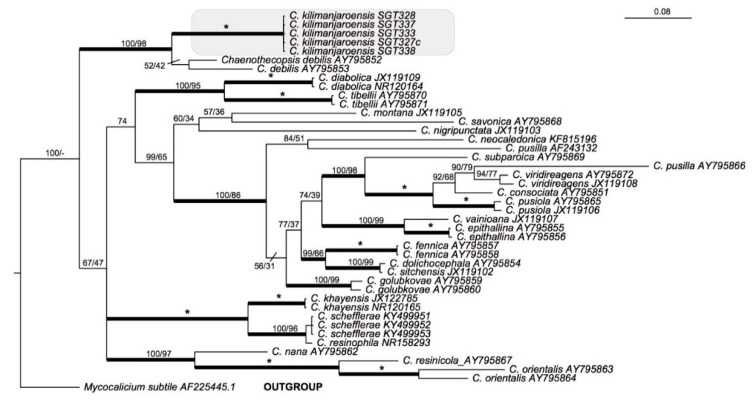
Phylogenetic relationships of 24 species of *Chaenothecopsis* based on a Bayesian analysis of an ITS dataset. The tree was rooted using *Mycocalicium subtile*. The two support values associated with each internal branch correspond to posterior probabilities (PP) and maximum likelihood bootstrap support (MLbs) proportions, respectively. Branches in bold indicate a support of PP ≥ 95% and MLbs ≥ 70%. An asterisk on a bold branch indicates that this node has a support of 100% for both support estimates. *Chaenothecopsis kilimanjaroensis* is highlighted by a shaded box.

**Phylogenetic position**: Bayesian and maximum likelihood analyses of 43 nuITS sequences representing 24 species of *Chaenothecopsis* with *Mycocalicium subtile* as an outgroup (Table 1), is presented in Figure 3. The outgroup was chosen with reference to the phylogeny [9]. Five sequences of *Chaenothecopsis kilimanjaroensis* have as a clade strong support in both analyses (PP = 1, MLbs = 100) and the most closely related species is *Chaenothecopsis debilis*.

**Note.** Like some other species in *Chaenothecopsis*, e.g., *C. consociata* (Nádv.) A.F.W. Schmidt, *C. epithallina* Tibell and *C. formosa* Titov, this species is a parasite/commensal on lichen thalli, which eventually seems to kill the host thallus. It is unusually variable in its ascoma morphology and the apothecia seem to either develop to being relatively long-stalked and carrying one capitulum only, or, alternatively, several more or less aggregated capitula are formed on a short stalk. When well-developed the stalk is distinctly pale olivaceous brown and more or less translucent in water-mount, a feature very unusual in the genus. *Chaenothecopsis pusilla* (Ach.) A.F.W. Schmidt has occasionally been observed to have a stalk that is rather pale towards the base, but then usually with a greenish or greenish blue tinge [22]. In having irregularly aggregated capitula it also recalls *C. amurense* Titov [10] but differs i.e., in host (*Trentepohlia* for the latter) and in having 1-septate, rather than non-septate, spores. It might be noted that *C. pusilla* is not monophyletic in the analysis. This species as currently construed probably harbors a complex of morphologically similar but genetically different species that have not yet been studied in detail.

***Chaenothecopsis pilosa* Tibell & Kalb**

**Habitat**: A commensal/parasite on thalli of *Tylophoron moderatum* in montane cloud forests.

**Distribution**: Widely distributed in the tropics. Described from Mt. Meru, Arusha region [53]. Further known from Papua New Guinea, Guatemala, Peru and Brazil.

***Heterocyphelium leucampyx* (Tuck.) Vain.**

**Specimen examined**: **Tanzania**, Alstrup Tz2165 (UDSM).

**Habitat**: Collected at 625 m altitude ion the Udzungwa mountains in Morogoro.

**Distribution**: Reported from the Morogoro Region (Udzungwa Mts.) [41], but from Africa previously known from the Ivory Coast [21]. Additionally occurring in Australia and South America.

**Note**: The unusual features and problematic placement of *Heterocyphelium* among the calicioids have earlier been pointed out [7] and recently it has, based on sequence data, been shown to belong in Arthoniales [54].

***Mycocalicium victoriae* (F. Wilson) Tibell**

Reported from Tanzania and Madagascar [21].

**Habitat**: On wood in montane cloud forests.

**Distribution**: Originally described from Australia this species is now also known from New Zealand, North America, and Europe.

***Pyrgillus cambodiensis* Kashiw., K.H. Moon &Aptroot**

**Specimen examined**: **Tanzania**, Morogoro Region, Morogoro District, Uluguru Mts., above Tschenzema village, 07°06’45’’ S, 37°36’45’’ E, elev. 2400–2450 m, 1999 Frisch 99/Tz147 (hb. Frisch); 07°06’45’’ S, 37°36’45’’ E, alt. 2400–2450 m, Frisch 99/Tz1896 (hb. Frisch); Frisch 99/Tz2811 (hb. Frisch).

**Habitat**: In mountain rainforest at moderate altitudes.

**Distribution**: Originally described from Cambodia [55] and also known from China. This species is here for the first time reported in Africa.

**Note.** Tanzanian material has previously been reported as *Pyrgillus javanicus* [41]. The species was discussed in [55]. New to Africa.

***Pyrgillus javanicus* (Mont. & Bosch) Nyl.**

**Habitat**: In moderate altitudes of mountain cloud forests and on bark of old tree in open mountain rainforest.

**Distribution**: Reported from the Tanga Region (E. Usambara Mts.) by [21], and from the Morogoro Region (Uluguru Mts.) by [41]; see under *P. cambodiensis*).

**Note.** The species has also been reported from Madagascar and Kenya [21].

***Sphinctrina tubaeformis* A. Massal.**

First recorded from Tanzania by [56] from the Usambara Mts.

**Habitat**: Occurring as a commensal/parasite on *Pertusaria.*

**Distribution**: Widely distributed in tropical to warm temperate areas, also known from North America, Australasia, Africa and Europe.

***Tylophoron moderatum* Nyl.**

**Specimens examined**: **Tanzania**, Iringa, Ilutila village, Alstrup 649, 08°15’48.87’’ S, 35°57’20.98’’ E, elev. 1423 m (UDSM).

**Habitat**: On bark of smooth-barked trees in mountain rain forests at low or moderate altitudes.

**Distribution**: First reported from Tanzania in [56] and further in [37] when *T. moderatum* var. *modestius* Zahlbr. from the Usambaras was described. A further record (Arusha) was given by [21].

***Tylophoron protrudens* Nyl.**

Reported from Mt. Meru and Ngurdoto (Arusha region) by [21] and Iringa district, Udzungwa Mts. (Ilutile) by [40].

**Habitat**: On bark of smooth-barked trees in mountain rain forests.

**Distribution**: Widely distributed in tropical/subtropical areas and known also from Rwanda and Zaire [39] and further Guinea and Kenya [21].

## 4. Discussion

Twenty-six (26) crustose calicioids reported here occur in Tanzania, most of them in mountain cloud forests, but some lowland species have also been included to make the list of crustose calicioids of Tanzania complete. Most species belong to *Calicium*, *Chaenotheca*, and *Chaenothecopsis*.

### 4.1. Habitats of Cloud Forest Crustose Calicioids

Calicioids have been shown to occur mainly in undisturbed forests with a long forest continuity [11,57], and they have frequently been used as bioindicators of habitats with high species diversity and as indicators of “key habitats” [12]. In addition, many are included in Red lists of several countries [58,59]. In Tanzania this pattern is also clearly discernable and calicioids mainly occur in mountain cloud forests (middle and upper forest zones) and their niches are found on the bark of old trees and on lignum.

### 4.2. Distribution of Tanzanian Cloud Forest Crustose Calicioids

Most of the Tanzanian crustose calicioids belong to a group of species with wide distributions in cool areas of both hemispheres and occasional occurrence in high mountains of low latitudes. Consequently, they form an important biota in mountain cloud forests and deserve attention in the context of preserving biodiversity and developing conservation policies. The wider distribution of Tanzanian cloud forest calicioids may be detailed on several levels. On the most general level two patterns may be discerned:

#### 4.2.1. Species with a Main Distribution in Cool Temperate Areas—“Temperate Species”

Many of the species are widely distributed, with high frequencies in temperate to cool temperature areas of both hemispheres, as well as at high altitudes in mountains at lower latitudes. Thus several are, apart from on the high mountains of Africa, also found along the Andes, but at low latitudes only in its high mountains, e.g., in Equador and Venezuela [27]. Similarly, some of these species occur in high altitudes along the Himalaya and its continuation to the Tasman Belt, e.g., in Papua-New Guinea [31]. *Calicium* species, such as *C. indicum* Tibell, *C. laevigatum* Tibell, *C. nobile* Tibell, *C. pyriforme* Tibell, *C. tenuisporum* Tibell and *C. verrucosum* Tibell, are representatives of this group.

In Africa occurrences of this “cool temperate element” are found from the Atlas, at high altitudes of the African volcanoes and the East Arch Mountains down to lower altitudes in Southern Africa [21]. Some of these distributions nowadays appear very disjunct, but dispersal might have happened during Ice Age temperature depressions and associated widening of areas with temperate forests over large parts of Africa in connection with the quaternary glaciations.

The following species are good African representatives: *Calicium abietinum* Pers., *C. lenticulare* Ach., *C. salicinum* Pers., *Chaenotheca chrysocephala* (Ach.) Th. Fr., *C. furfuracea*, *C. hispidula*, *C. stemonea*, *C. trichialis*, and *Chaenothecopsis debilis*.

#### 4.2.2. Species with Main Occurrences in Warm Temperate to Tropical Areas—“Pantropical Species”

The other type of distributions encountered among Tanzanian cloud forest calicioids comprises species with an often wide distribution in warm temperate to subtropical areas. Here belong, for example, *Calicium hyperelloides*, *Chaenotheca olivaceorufa*, *Chaenothecopsis pilosa*, *Heterocyphelium leucampyx*, *Mycocalicium victoriae*, *Pyrgillus javanicus*, *Sphinctrina tubaeformis*, *Tylophoron moderatum,* and *T. protrudens.*

## Figures and Tables

**Table 1:** Specimens used in an Internal transcribed spacer (ITS)-based phylogenetic analysis of *Chaenothecopsis* species together with their GenBank accession numbers. New sequences are in bold.

Species	Country	Voucher	GenBank Accession No.
*C. consociata*	Sweden	Tibell 22472	AY795851
*C. debilis*	New Zealand	Tibell 16643	AY795852
*C. debilis*	Sweden	Tibell 22796	AY795853
*C. diabolica*	USA	H:Tuovila 06-035	JX119109
*C. diabolica*	USA	H:Tuovila 06-035	NR.120164
*C. dolichocephala*	Russia	Tibell 19281	AY795854
*C. epithallina*	Sweden	Tibell 22705	AY795855
*C. epithallina*	Sweden	Tibell 22793	AY795856
*C. fennica*	Sweden	Tibell 16024	AY795857
*C. fennica*	Sweden	Tibell 22718	AY795858
*C. golubkovae*	China	Titov 6707	AY795859
*C. golubkovae*	India	Tibell 23211	AY795860
*C. khayensis*	Ghana	H:JR 04G058	JX122785
*C. khayensis*	Ghana	H:JR 04G058	NR.120165
*C. kilimanjaroensis*	Tanzania	SGT 327c	**MN575663**
*C. kilimanjaroensis*	Tanzania	SGT 328	**MN575660**
*C. kilimanjaroensis*	Tanzania	SGT 333	**MN575662**
*C. kilimanjaroensis*	Tanzania	SGT 337	**MN575661**
*C. kilimanjaroensis*	Tanzania	SGT 338	**MN575664**
*C. montana*	Finland	H:Tuovila	JX119105
*C. nana*	Sweden	Tibell 22473	AY795862
*C. neocaledonica*	New Caledonia	Rikkinen 010179	KF815196
*C. nigripunctata*	USA	H:Tuovila 06-013	JX119103
*C. orientalis*	Russia	Tibell 19371	AY795863
*C. orientalis*	India	Tibell 23216	AY795864
*C. pusilla*	New Zealand	Tibell 16580	AF243142
*C. pusilla*	Sweden	Tibell 22804	AY795866
*C. pusiola*	Sweden	Tibell 15884	AY795865
*C. pusiola*	Finland	H:Tuovila 09-047	JX119106
*C. resinicola*	Russia	Tibell 19234	AY795867
*C. resinophila*	China	H:JR 000424	NR.158293
*C. savonica*	Sweden	Tibell 15876	AY795868
*C. schefflerae*	New Zealand	Beimforde 040	KY499951
*C. schefflerae*	New Zealand	Beimforde 047	KY499952
*C. schefflerae*	New Zealand	Beimforde 049	KY499953
*C. sitchensis*	USA	H:Tuovila 06-033	JX119102
*C. subparoica*	Italy	Tretiach (hb. Tretiach)	AY795869
*C. tibellii*	China	Titov 6655	AY795870
*C. tibellii*	China	Titov 6662 (LE)	AY795871
*C. vainioana*	Finland	H:Tuovila 09-066	JX119107
*C. viridireagens*	Finland	H:Tuovila 09-068	JX119108
*C. viridireagens*	Sweden	Tibell 22803	AY795872
*Mycocalicium subtile*	Sweden	Tibell 21020	AF225445
